# “Out of office”: Availability norms and feeling burned out during the COVID-19 pandemic: The mediating role of autonomy and telepressure

**DOI:** 10.3389/fpsyg.2023.1063020

**Published:** 2023-02-21

**Authors:** Karolien Hendrikx, Joris Van Ruysseveldt, Karin Proost, Sandra van der Lee

**Affiliations:** ^1^Department of Work and Organizational Psychology, Open Universiteit, Heerlen, Netherlands; ^2^Faculty of Economics and Business, KU Leuven, Belgium

**Keywords:** social norms, availability norms, ICT use, job autonomy, telepressure, burnout symptoms

## Abstract

**Introduction:**

Digital innovations make it possible to work anywhere and anytime using any kind of device. Given these evolutions, availability norms are emerging at work. These norms specifically refer to the experienced beliefs or expectations from colleagues or superiors to be available for work-related communication after regular work hours. We rely on the Job-Demands Resources Model as we aim to investigate the relationship between these availability norms and burnout symptoms during the COVID-19 pandemic. We first of all study to what extent availability norms are associated with heightened burnout symptoms. Secondly, we study how both a personal demand, namely telepressure, and a job resource, namely autonomy, could offer distinct and relevant explanations for the role these availability norms play in experiencing burnout symptoms.

**Method:**

We collected data through a survey study with 229 employees from various organizations in the second half of 2020.

**Results:**

The findings indicated that indeed availability norms are significantly associated with more burnout symptoms and that both heightened telepressure and reduced autonomy mediated this relationship.

**Discussion:**

This study contributes to theory and practice as we offer insight into how availability norms at work could be detrimental for the occupational health of employees, which can be taken into account when setting up rules and regulations at work.

## 1. Introduction

Remote information exchange and interaction with co-workers are becoming increasingly rich and efficient because of modern Information and Communication Technology (ICT) (Allen et al., 2015). Work can take place independent of time and place, which implies that, for many employees, work is no longer restricted to the traditional work hours at the office. Various devices increasingly enable employees to work anywhere/anytime, blurring boundaries with other life domains (Robelski and Sommer, 2020; Hassard and Morris, 2022). The COVID-19 pandemic gave this development an important boost, as many employees were required to work from home (Graham et al., 2021; Nimrod, 2022).

Several studies show how ICT developments during the past decades come at a cost of heightened stress and burnout risks (Day et al., 2012; Salmela-Aro and Upadyaya, 2018; Karimikia et al., 2021). Burnout, a work-related state “characterized by extreme tiredness, reduced ability to regulate cognitive and emotional processes, and mental distancing” (Schaufeli et al., 2020, p. 4), affects more than 10% of employees across job types and sectors (Demerouti et al., 2021). Especially the emergence of availability norms at work deserves more attention as a potential risk factor associated with burnout symptoms. These norms concern perceived implicit beliefs or expectations from colleagues or superiors (or other influential people) regarding the availability for work-related asynchronous communication after work hours: Do my colleagues and superiors expect me to respond fast to work-related messages after work hours, or can I wait until regular work hours to process these messages? Worries about these implicit availability norms have prompted various policymakers to set up more explicit regulations regarding work-related communication after work hours. For instance, a recent law in Belgium states that government workers do not have to answer their bosses’ e-mails after work hours (Treisman, 2022). But what exactly is the impact of implicit availability norms on employees’ wellbeing? Are these norms related to burnout symptoms and if so, what are relevant underlying mechanisms?

In this study, we focus on the relationship between availability norms regarding off-job ICT use and burnout symptoms. We specifically address two research questions. First, what is the relationship between these availability norms and burnout symptoms? Secondly, how can we explain the relationship between availability norms and burnout symptoms? Building on the Job Demands Resources Model (JDR Model, Bakker and Demerouti, 2007), we study the mediating role of one relevant demand, namely telepressure (Barber and Santuzzi, 2015), and of one relevant resource, namely job autonomy (Hackman and Oldham, 1980). By doing this, we answer to the call of Schlachter et al. (2018) to see more research investigating the mediators in the relationship between these kind of availability norms and wellbeing at work. We also follow previous research that highlighted how ICT usage offers important opportunities for organizing work adaptively, heightening flexibility and autonomy (Sardeshmukh et al., 2012; Mazmanian et al., 2013; Spreitzer et al., 2017). At the same time, it entails threats in the form of heightened (tele)pressure to do the work immediately from wherever you are (Barber and Santuzzi, 2015). This dual impact of ICT use has been referred to as the autonomy paradox (Mazmanian et al., 2013). Job autonomy, the first mediator, generally refers to the extent to which one can determine the method of work (Hackman and Oldham, 1980). In this study, the concept of autonomy specifically focusses on being able to determine the timing and pace of work. Within the JDR Model, job autonomy is considered a job resource with the potential to motivate employees and reduce strain (Bakker and Demerouti, 2007; Sardeshmukh et al., 2012). The role of autonomy in relation to work-related availability has often been raised in the literature, but is much less studied (Thörel et al., 2022). Telepressure, the second mediator, is defined as feeling an urge to be responsive to others through ICT-driven communication with the preoccupation that one must respond quickly (Barber and Santuzzi, 2015). Previous studies on the concept of telepressure show a link between experienced telepressure and burnout symptoms (Barber and Santuzzi, 2015, 2017). Within the JDR Model (Bakker and Demerouti, 2007), telepressure can be considered a personal demand that induces strain at work.

In this paper, we will argue that availability norms thwart employees’ autonomy and instigate (tele)pressure, so that employees are more inclined to feel burned out at work. Relying on survey data of 229 employees from various organizations collected during the autumn of 2020, we investigate the relationship between availability norms and burnout symptoms as well as the mediating mechanisms of job autonomy and telepressure.

This study contributes to the literature in two relevant ways. First, rather than focusing on extended availability for work in general (Cooper and Lu, 2019) or on actual ICT use after work (Schlachter et al., 2018), we focus on the associated availability norms as a temporally and spatially defined aspect related to work-related extended availability. Studying how these specific norms relate to burnout symptoms can give concrete, actionable insight into the risks associated with these implicit availability norms. After all, these insights raise awareness for paying deliberate attention to which implicit norms develop in organizations. Second, we add to these insights on availability norms by studying the role of two relevant mediators. While the association between availability norms and telepressure has been investigated, empirical studies that address the relationship with job autonomy are limited (Thörel et al., 2022). By investigating the mediating role of telepressure in combination with job autonomy, we contribute to theory and practice as we offer insight into relevant psychological processes associated with availability norms at work. These insights can guide setting up explicit principles and regulations at work that respect autonomy and reduce telepressure as a means to develop a social-normative context wherein occupational health is safeguarded.

## 2. Theoretical framework

### 2.1. Availability norms and burnout

Social influence theory suggests that the social environment motivates us to adapt our behavior according to salient social norms (Cialdini and Goldstein, 2004). Social norms help us gain insight in and effectively respond to social situations. As people are fundamentally driven to build meaningful relations with others, gaining approval of others is important. As such, people generally believe that acting according to what is seen as the “right behavior” in a given situation, will provide that approval (Cialdini and Goldstein, 2004). Hence, social norms guide action. Cialdini et al. (1990) argued that the impact of norms on people depends on the type of norm and on the situation. Social norms can either be descriptive (what is typical or normal behavior) or prescriptive (what ought to be or what is desirable behavior) and they are generally dependent on the specific setting. In a work setting, social norms can be seen as a set of unwritten rules of conduct of a group of colleagues (Elster, 1989), which can encompass various domains. For instance, there can be social norms regarding taking breaks, holding meetings, or using ICT tools.

New ICT tools give rise to opportunities to interact with colleagues in more diverse ways. These digitalized interactions introduce new habits, and these habits then become reciprocal expectations (Diekmann and Przepiorka, 2016) and steadily, social availability norms are beginning to arise. We specifically define these availability norms as perceived implicit rules regarding being available for work-related communication after regular work hours. In the context of technology usage, social norms may arise from interactions with peers or with the supervisor (Taylor and Todd, 1995; Morris and Venkatesh, 2000). Specifically, availability norms emerge if employees see colleagues responding quickly to e-mail outside office hours and they also start to do so in order to feel accepted and part of the group. Between colleagues, it will be considered as “normal” to respond to work-related messages outside working hours, as “everybody does it.” Or availability norms arise when employees believe that their supervisor expects a quick response and they would want to live up to those expectations. Expectations of peers as well as supervisors thus play an important role in the development of social norms for responding to work-related messages outside working hours. In this regard, availability norms are closely related, yet distinct from the concept of extended availability requirements (Dettmers et al., 2016) in the sense that the latter entail formal as well as informal requirements from the organization while the former solely concerns the informal expectations from key persons in the immediate social environment. Another related concept are the perceived segmentation norms of Derks et al. (2014). This concept addresses the workplace norms regarding the degree in which one can mentally segment work and home life, which is a broader concept compared to availability norms. Availability norms specifically concern the norms regarding availability for work-related asynchronous communication, which is only one concrete example of a means to segment work and home life.

In this paper, we assume these availability norms to be related to burnout symptoms. Schaufeli et al. (2020) specifically define burnout as a state characterized by exhaustion, cognitive and emotional deregulation, and mental distancing. It concerns a psychological syndrome resulting from chronic stressors in the social environment at work. Availability norms would instigate such a stressor. As socially embedded norms of conduct guide behavior (Ajzen, 1991), in teams where availability norms emerge, employees will feel inclined to respond quickly to messages outside work hours. This would effectively lead to spending more time replying to e-mails or other work-related messages after regular hours. In this respect, previous research found that these norms increased work-home interference (Derks et al., 2015; Gadeyne et al., 2018), which has been shown to be strongly linked with feeling burned out at work (Derks and Bakker, 2014). Availability norms are thus expected to trigger an enduring stressor, heightening the risk of burnout complaints at work. Therefore, we hypothesize:

*Hypothesis 1*: Availability norms are positively related to burnout symptoms.

### 2.2. The role of telepressure as demand

Because availability norms are expected to be associated with a higher risk of experiencing burnout, it is important to gain further insight into the underlying processes. Are these social norms detrimental because they put individuals under pressure to be constantly responsive? Barley et al. (2011) showed, relying on qualitative and quantitative data, that norms related to availability can lead to increased use of ICT in the evening and weekends. Respondents in their study explicitly mentioned that they felt a pressure coming from experienced expectations within the organization. Respondents said they had the feeling that they could not easily ignore email and other work-related messages. In line with this, Barber and Santuzzi (2015, 2017) introduced the concept of telepressure, referring to the urge to respond quickly to work-related messages. It specifically involves pressure associated with asynchronous ICT-based communication. Telepressure is different from actual ICT use after work hours, as this ICT use is the behavior often (but not necessarily) associated with telepressure. In this regard, Barber and Santuzzi (2015) define telepressure as a psychological state rather than ICT behavior. They demonstrated that there is an important correlation between social norms and telepressure. Specifically, prescriptive norms seem to be relevant predictors herein. According to Barber and Santuzzi (2015), an employee who experiences telepressure has internalized the (external) availability norms. In other words, availability norms concern the external socially constructed expectations that have the potential to become internalized by the individual in the form of telepressure.

Telepressure leads to employees being “connected to work” for longer periods of time, responding quickly to messages also outside regular working hours (Barber and Santuzzi, 2015). The JDR Model considers demands as physical, psychological, social, and organizational aspects that require physical or psychological effort and are therefore associated with physical and/or psychological costs (Schaufeli and Taris, 2014). Telepressure is a psychological aspect that provokes psychological effort and thus meets the definition of demand. It specifically entails a personal demand, which refers to “the requirements that individuals set for their own performance and behavior that force them to invest effort in their work and are therefore associated with physical and psychological costs” (Bakker and Demerouti, 2017, p. 279). Prolonged demand exposure sets a health impairment process in motion which leads to a decrease in mental energy, which can ultimately result in health problems and burnout (Schaufeli and Bakker, 2004; Bakker and Demerouti, 2007).

Barber and Santuzzi (2015, 2017) indeed found that people who experience telepressure more often show symptoms of burnout. Moreover, various other studies revealed important negative wellbeing effects of telepressure. For instance, high telepressure is associated with less psychological detachment from work (Barber and Santuzzi, 2015, 2017; Van Laethem et al., 2018), less sleep (Barber and Santuzzi, 2015, 2017; Van Laethem et al., 2018; Barber et al., 2019; Cambier et al., 2019; Rogers and Barber, 2019), more absenteeism (Barber and Santuzzi, 2015), and a poorer work-family balance (Grawitch et al., 2018; Barber et al., 2019).

**Figure 1:**
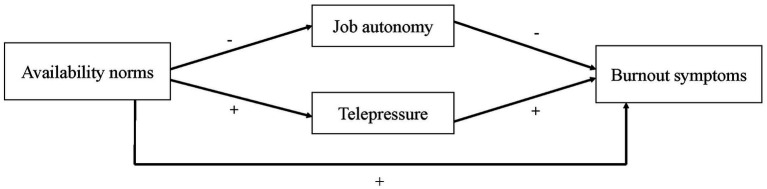
Research model.

As previous research demonstrated that availability norms are positively related to telepressure and that telepressure is positively related to experiencing burnout symptoms, we argue that telepressure is a relevant factor explaining the relationship between availability norms and burnout symptoms (see Figure 1). This leads to the following hypothesis:

*Hypothesis 2*: As availability norms are positively related to telepressure (H2A) and telepressure is positively related to feeling burned out (H2B), telepressure mediates the relationship between availability norms and burnout (H2C).

### 2.3. The role of autonomy as resource

Research consistently shows that job autonomy is positively associated with employee wellbeing (Clausen et al., 2022; Ryu et al., 2022). Autonomy is considered to be a job resource within the JDR Model. Job resources specifically concern those physical, social, or organizational aspects of work that not only may reduce the physiological and psychological costs of job demands, but also facilitate the achievement of work goals and stimulate thriving at work (Bakker and Demerouti, 2007). Research has shown that autonomy as job resource increases engagement and performance, and reduces the risk of feeling burned out (Schaufeli and Bakker, 2004; Schaufeli et al., 2009; Bakker and Demerouti, 2017). While job autonomy is generally considered as a relatively stable work characteristic (Brauchli et al., 2013), evidence shows that the experience of autonomy can fluctuate depending on changes in the work situation (Petrou et al., 2012).

As ICT tools offer opportunities to organize work flexibly, they hold the potential for increasing the job autonomy employees’ experience (Mazmanian et al., 2013; Ninaus et al., 2015). Yet, Mazmanian et al. (2013) discuss how ICT developments could at the same time also limit autonomy. Because ICT makes it possible to work anytime and anywhere, co-workers or superiors could start to expect that you are indeed always and everywhere available for work. In other words, increased opportunities to organize work flexibly according to your personal needs could rapidly shift to increased expectations to constantly stay connected for work. This has been referred to as the autonomy paradox (Mazmanian et al., 2013).

As availability norms can be seen as perceived expectations for replying to work-related messages without delay, we argue that precisely these availability norms could be responsible for undermining autonomy. Previous research showed that social norms that involve expectations to stay connected after work hours have a detrimental impact on work-family boundary dynamics (Derks et al., 2014; Gadeyne et al., 2018). It is argued that they reduce the boundary control that employees experience (Kossek and Lautsch, 2012; Allen et al., 2014; Schieman and Glavin, 2017). Employees develop the feeling that they are no longer in control of how they organize their work schedules in relation to their private lives. In other words, these norms are associated with lower experienced job autonomy.

Relying on the Self-Determination Theory in combination with the JDR Model, Fernet et al. (2013) demonstrated how lower experienced job autonomy is related to higher burnout symptoms. According to Self-Determination Theory, experiencing autonomy is one of the three basic psychological needs, alongside experiencing competence and relatedness. Work aspects that fulfill these needs can be seen as psychological resources that energize behavior. When these needs are thwarted, as is the case with availability norms undermining employees to feel control over their work schedules (i.e., undermining the need for autonomy), psychological wellbeing is likely to decline (Deci and Ryan, 2008). In this regard, a lack of discretion and experienced freedom when doing a job has been associated with burnout symptoms (Van den Broeck et al., 2008; Fernet et al., 2013). Specifically in relation to extended availability for work, previous research showed how non-autonomous motivation for extended work is associated with various indicators of reduced wellbeing (Cooper and Lu, 2019). To sum up, we argued that availability norms are expected to be negatively related to job autonomy and demonstrated that previous research showed that low autonomy is related to experiencing higher burnout symptoms. Therefore, we assume that job autonomy is a relevant factor explaining the relationship between availability norms and burnout symptoms. Hence, we hypothesize that:

*Hypothesis 3*. As availability norms are negatively related to autonomy (H3A) and autonomy is negatively related to feeling burned out (H3B), autonomy mediates the relationship between availability norms and burnout (H3C).

## 3. Method

### 3.1. Procedure and participants

The respondents for this study were recruited through one of the author’s professional network in the Netherlands during the autumn of 2020. It is relevant to note that at that time there were strict COVID-19 regulations requiring working from home as much as possible. Respondents were contacted *via* e-mail and social media, and could immediately click through to the survey in the LimeSurvey tool. It was important that participants from both private and public organizations were engaged, as social norms for ICT use outside working hours could vary considerably across organization types.

A number of inclusion criteria for participation were held. First, the employee had to have access to and be able to use the organization’s ICT outside regular working hours. Second, participating employees had to be in the age range of 18 to 67 years to represent the active working population. Third, the participating employees were required to have a contract of 24 h or more per week. We opted for 24 h to make sure that the respondent would be working a considerable part of the week. These criteria were communicated before the survey started. Respondents who did not meet the criteria were removed from the data set. We also excluded questionnaires that were not fully completed.

A total of 293 respondents took part in the study, 229 of whom fully completed the questionnaire. Descriptives of the participants are depicted in Table 1. The respondents ranged in age from 20 to 66 years with an average age of 46 years. 48.5% of the respondents were male and 51.1% were female. In addition, 0.4% of the respondents indicated to be of a different gender or did not answer this question. Most of the respondents (86.5%) had a higher level of education (bachelor or master’s degree), which is in line with what can be expected of employees who are able to use work-related ICT outside work hours. 63.3% of the respondents worked for the (semi) public sector and 36.7% in private, commercial companies. Working hours per week (based on employment contract) varied between 24 and 40 h with an average of 35 h per week. Respondents also reported working outside of this contractual time: On average 6 h per week. At the time of measurement, 5.2% of respondents did not work from home at all, 29.3% worked partially from home and 65.5% worked completely from home, due to COVID-19 regulations.

**Table 1:** Participant descriptives.

Variable	Categories	Frequencies	Ratio
Gender	Male	111	48.5
	Female	117	51.1
	Other	1	0.4
Sector	Government	145	63.3
	Private company	84	36.7
Education	Secondary education	31	13.5
	Bachelor	101	44.1
	Master	97	42.4
Home office	Full-time	150	65.5
	Part-time	67	29.3
	No	12	5.2
		**Mean**	**SD**
Age		46.08	11.16
Hours by contract		35.38	4.26
Workhours outside contract		5.72	7.36

### 3.2. Measures

Availability norms. The availability norm scale that was used in this study was adapted from the “subjective expectation of technology use” of Venkatesh and Davis (2000), which consisted out of two items. Since the original measure was created in relation to the acceptance of new technology inside the workplace, items were modified to reflect on replying to work-related messages outside work hours. Moreover, rather than a strict prescriptive wording referring to what “I” as the respondent is expected to do, we rephrased toward a more neutral wording referring to what one finds important regarding work-related messages outside work hours. Specifically, the item “People who are important to me think I should use the system” was modified to “People who are important to me think you should keep up with work-related messages after work hours.” The item “People who have influence on my behavior think I should use the system” was modified to: “People who have influence on my behavior think that you should keep up with work-related messages after work hours.” Also, the following two items were added to make sure respondents also deliberately reflect on the specific role of colleagues and the immediate supervisor as key persons at work: (1) Colleagues expect me to respond to work-related messages after work hours, and (2) My supervisor expects me to respond to work-related messages after work hours. With the two extra items, the scale consists of four items. Responses were given on a 5-point Likert scale (1 = strongly disagree to 5 = strongly agree). Cronbach’s alpha for this scale is 0.92 and an exploratory factor analysis with all the key variables under study revealed the availability norms scale as a clearly distinct construct.

Telepressure. Telepressure was measured with an 8-item scale developed by Barber and Santuzzi (2015). The items were preceded by the following instruction: “For the following questions, think about how you use technology to communicate with people for work activities outside regular work hours. Think especially of ICT that you can use time-independently (email, text messaging, etc.). Please indicate to what extent you agree or disagree with the statements.” Responses were given on a 5-point Likert scale (1 = strongly disagree and 5 = strongly agree). A sample item is: “I cannot stop thinking about a message until I’ve answered it.” Cronbach’s alpha for this scale is 0.90.

Job autonomy. Job autonomy was measured with 4 items of the NOVA WEBA questionnaire (Dhondt and Houtman, 1997), specifically focusing on the temporal dimension of autonomy. In the original questionnaire, questions had to be answered with “yes” or “no.” In this study, the questions were rephrased into statements in order to be able to add more nuance to the answers. These items are scored on a 5-point Likert scale (1 = totally disagree and 5 = totally agree). Also, one item has been changed as follows: “If necessary, I can postpone response time to work-related messages.” An example of another statement is: “I can decide for myself when I perform a certain task.” Cronbach’s Alpha for this scale is 0.85.

Burnout symptoms. Burnout symptoms were measured with the work-related version of the BAT (Burnout Assessment Tool) (Schaufeli et al., 2020). The BAT is a self-assessment questionnaire, consisting of four scales to measure exhaustion (eight items; α = 0.91), mental distancing (five items; α = 0.82), cognitive deregulation (five items; α = 0.86), and emotional deregulation (five items; α = 0.90), respectively. Responses were given on a 5-point Likert scale (1 = never to 5 = always). A sample item is: “During my work I get irritated easily when things do not go the way I want.” The items from the four scales were combined to produce a single outcome for burnout symptoms (α = 0.95).

Control variables. We first of all included gender and age as control variable, because they appear to be related to burnout (Purvanova and Muros, 2010; Gómez-Urquiza et al., 2017). Many burnout studies focused on specific occupational groups with varying educational backgrounds (e.g., nurses, teachers, etc.). Also the degree in which one works after work hours, appears to differ according to occupational type and level (Chung and Van der Horst, 2020). To account for these differences, we incorporated education (ranging from primary education (1) to master’s degree (5)) and sector (Government (1) or Private (2)) as control variables. We also included the number of hours worked outside work hours on ICT tools in an average work week, because work hours are also related to feeling burned out (Park and Lake, 2005; Lin et al., 2021). Lastly, as the survey took place in 2020, many employees were required to work from home due to COVID-19 restrictions. Therefore, we also included whether respondents worked at the workplace (1), in a hybrid form (2), or exclusively from home (3).

### 3.3. Analysis

We first of all present the descriptive indicators Cronbach alpha’s, means, standard deviations, and correlations. As an additional preliminary analysis, the results of an exploratory factor analysis will be presented. Our hypothesis testing will take place in three steps. First, we perform a hierarchical linear regression, for the main effects of availability norms, job autonomy, and telepressure, respectively, on burnout symptoms. Second, we study the main effects of availability norms on job autonomy and telepressure, through two multiple linear regression analyses. Third, we study the potential mediation effect of job autonomy and telepressure in explaining the relationship between availability norms with burnout symptoms using the PROCESS 2.13 Macro of Hayes and Rockwood (2017) in SPSS.

## 4. Results

### 4.1. Preliminary analyses

Means, standard deviations, and correlation coefficients of the variables under study are presented in Table 2. An exploratory factor analysis (principal component analysis; PCA) with Varimax rotation was performed to investigate whether the relevant constructs under study could be meaningfully distinguished from each other. Seven components were extracted (Eigenvalue above 1; Factor loadings for all items above 0.45), representing availability norms, telepressure, job autonomy, and the four burnout symptoms scales.

**Table 2:** Cronbach alpha’s, means, standard deviations and correlations among the study variables (*N* = 229).

	α	*M*	SD	1	2	3	4	5	6	7	8	9
1. Availability norms	0.92	2.16	0.87									
2. Job autonomy	0.85	4.02	0.61	−0.31**								
3. Telepressure	0.9	2.78	0.83	0.47**	−0.30**							
4. Burnout symptoms	0.95	2.22	0.55	0.23**	−0.33**	0.33*						
5. Gender	NA	1.49	0.51	0.09	−0.03	−0.03	−0.10					
6. Age	NA	46.08	11.16	−0.06	−0.09	−0.07	−0.03	0.11				
7. Education	NA	4.21	0.89	−0.06	0.07	−0.05	0.03	−0.22**	−0.19**			
8. Sector	NA	1.37	0.48	0.16*	0.07	0.05	−0.08	0.17**	−0.20**	−0.02		
9. Work after hours	NA	5.72	7.36	0.09	0.08	0.10	−0.01	0.14*	0.16*	−0.09	0.04	
10. Work from home	NA	2.6	0.59	−0.16*	−0.01	−0.06	0.14*	−0.09	0.08	0.15*	−0.29**	−0.03

*p ≤ 0.05; **p ≤ 0.01.

### 4.2. Hypothesis testing

Main effects. Hypotheses 1 stated that availability norms are positively related to burnout. To test this hypothesis, we set up a multiple linear regression analysis. Model 2 in Table 3 demonstrates that indeed we found a significant main effect of availability norms (*β* = 0.281, *t*(221) = 4.328, *p* < 0.001) on burnout symptoms, which offers support for Hypothesis 1.

**Table 3:** Multiple linear regression of Burnout symptoms on predictor variables.

Step	Variables entered	Model 1	Model 2	Model 3
1	Gender	−0.091	−0.107	−0.087
	Age	−0.046	−0.033	−0.041
	Education	−0.055	−0.054	−0.025
	Sector	−0.039	−0.069	−0.042
	Work after hours	0.015	−0.006	0.002
	Work from home	0.153*	0.187**	0.180**
2	Availability norms		0.281***	0.098
3	Telepressure			0.227**
	Job autonomy			−0.224***		*R*^2^ = 0.037	*R*^2^ = 0.113	*R*^2^ = 0.212

Standardized regression coefficients are reported. *p ≤ 0.05; **p ≤ 0.01; ***p < 0.001.

Hypothesis 2 addressed the role of telepressure in the relationship between availability norms and feeling burned out. We first investigated the main effects through multiple linear regression analyses. First, focusing on the relationship between telepressure and burnout symptoms, we found that telepressure is significantly positively related to burnout symptoms (*β* = 0.227, *t*(219) = 3.239, *p* < 0.01), which is in line with H2B (See Model 3 in Table 3). Second, Table 4 presents the main effect of availability norms on telepressure. As expected in line with H2A, availability norms appeared to be strongly positively related to experiencing telepressure (*β* = 0.470, *t*(221) = 7.803, *p* < 0.001).

**Table 4:** Multiple linear regression of Telepressure on predictor variables.

Step	Variables entered	Model 1	Model 2
1	Gender	−0.051	−0.077
	Age	−0.074	−0.053
	Education	−0.014	−0.013
	Sector	0.020	−0.031
	Work after hours	0.117	0.082
	Work from home	−0.080	−0.025
2	Availability norms		0.470***
		*R*^2^ = 0.027	*R*^2^ = 0.238

Standardized regression coefficients are reported. *p ≤ 0.05; **p ≤ 0.01; ***p < 0.001.

Hypothesis 3 concerned the role of job autonomy in the relationship between availability norms and feeling burned out. Again, we first examined the main effects through multiple linear regression analyses. Studying the main effect of job autonomy on burnout symptoms (See Model 3 in Table 3), we found that job autonomy is significantly negatively related to burnout symptoms (*β* = −0.224, *t*(219) = −3.396, *p* < 0.001), which is in line with H3B. Model 2 in Table 5 presents the main effect of availability norms on job autonomy. As expected in line with H3A, availability norms appeared to be negatively related to experiencing autonomy (*β* = −0.339, *t*(221) = −5.301, *p* < 0.001).

**Table 5:** Multiple linear regression of Job autonomy on predictor variables.

Step	Variables entered	Model 1	Model 2
1	Gender	−0.009	0.010
	Age	−0.073	−0.089
	Education	0.116	0.115
	Sector	0.053	0.089
	Work after hours	0.096	0.121
	Work from home	−0.014	−0.054
2	Availability norms		−0.339***
		*R*^2^ = 0.031	*R*^2^ = 0.141

Standardized regression coefficients are reported. *p ≤ 0.05; **p ≤ 0.01; ***p < 0.001.

**Figure 2:**
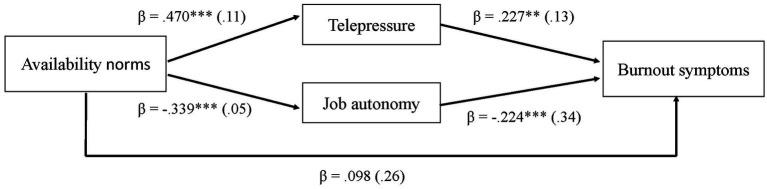
Mediation model for telepressure and job autonomy. Standard errors in parentheses. *p < 0.05; **p < 0.01; ***p < 0.001.

Mediational effects. To test the mediational effects of telepressure and job autonomy in the relationship between availability norms and burnout symptoms, we used Model 4 in Process (Hayes, 2017), by means of 5,000 bootstrapped samples. We included telepressure and job autonomy as two distinct parallel mediators. Burnout symptoms were included as dependent variable, availability norms as independent variable, and the control variables as covariates (*R*^2^ = 0.21). The analyses showed a significant indirect effect with telepressure as mediator (effect = 0.107, SE = 0.038, 95% CI = [0.037, 0.187]). As such, we found support for hypothesis 2. The analyses also showed a significant indirect effect with job autonomy as mediator (effect = 0.076, SE = 0.026, 95% CI = [0.031, 0.130]), which offered support for hypothesis 3. The full model is depicted in Figure 2.

## 5. Discussion

This cross-sectional study focused on the relationship between availability norms and burnout symptoms during the COVID-19 pandemic. We specifically examined how telepressure and job autonomy could explain this relationship, arguing that both play a distinct role in explaining how availability norms would pose a burnout risk. In line with these expectations, we indeed found that experiencing expectations to respond quickly to work-related messages after work hours, heighten the risk of burnout symptoms. This effect could be explained because of increased telepressure as well as decreased job autonomy.

### 5.1. Availability norms and burnout

In many work teams, there are availability norms emerging, referring to the perceived expectations to respond fast to work-related messages after work hours. To mitigate the potential health risks coming from these often implicit social norms, various policymakers are setting up explicit regulations regarding work-related messages after work hours, like prohibiting work-related e-mails after 6 PM. But in what way are these implicit norms to be constantly available effectively detrimental to the wellbeing of employees? The results in this study seem to suggest that the worries of these policymakers are legitimate, because we found that availability norms are indeed related to increased burnout symptoms at work. Previous research already linked availability norms with increased work-home interference (Derks et al., 2015; Gadeyne et al., 2018), which is associated with burnout symptoms (Derks and Bakker, 2014). We take it a step further, highlighting that these norms are associated with important health risks in the form of burnout symptoms. Moreover, the number of hours that an employee is effectively working with ICT after work hours appeared to not make a difference in experiencing burnout symptoms. Hence, it is not so much the after work hours *per se* but rather the associated social expectations that show a relationship with feeling burned out. Previous research by Kossek et al. (2012) showed that while employees differ in whether they prefer to integrate or strictly separate work and private life, what appeared to matter more than being a separator or an integrator is whether one feels in control of these boundaries. Our study is in accordance with this finding. Some people do not mind to work on e-mails after regular work hours, yet availability norms could undermine the important feeling of being in control in the form of heightened telepressure or reduced job autonomy.

### 5.2. Availability norms, telepressure, and burnout

The results indicated that there is a relationship between availability norms and experiencing telepressure. This was also found in previous studies wherein availability norms appeared an important predictor of the occurrence of telepressure (Barber and Santuzzi, 2015, 2017; Derks et al., 2015; Grawitch et al., 2018). Telepressure can be seen as a personal demand within the JDR Model, since it is a psychological job-related aspect that requires sustained effort. It precisely pressures individuals to be constantly responsive to work-related messages. Telepressure internalizes the availability norms that are experienced within a team. In line with previous research (Barber and Santuzzi, 2015, 2017), we also found that this telepressure comes with the assumed physical and/or psychological costs as it appeared positively related to burnout symptoms. Moreover, our study moves beyond this previous research, as we confirmed the expectation that heightened telepressure is a relevant mediator in the relationship between social availability norms and increased burnout symptoms.

### 5.3. Availability norms, job autonomy, and burnout

Besides telepressure, also the role of job autonomy in relation to ICT use after work has been discussed by scholars before. Mazmanian et al. (2013) presented the autonomy paradox referring to the ambiguous relation between work-related ICT use and experiencing autonomy at work. ICT on the one hand offers opportunities to work anytime and anywhere but it can also pose a threat as it can raise expectations to effectively work all the time and everywhere. Despite this discussion of the role of job autonomy in relation to work-related ICT-usage, research that explicitly studies the association with availability norms is lacking (Thörel et al., 2022). Therefore, in line with the reasoning offered by Mazmanian et al. (2013), we hypothesized that indeed these ICT-related expectations, i.e., availability norms, are associated with reduced job autonomy rather than the ICT use itself. Our results highlighted that this was the case, as we found an important negative relation between availability norms and experiencing autonomy at work.

Previous research indicated that reduced autonomy is associated with increased burnout risk (Schaufeli and Bakker, 2004; Schaufeli et al., 2009; Fernet et al., 2013; Bakker and Demerouti, 2017). Building on insights from Self Determination Theory and the JDR Model, this study confirmed that reduced experienced autonomy is related to heightened burnout symptoms. Adding further to the literature, we posited that job autonomy would be an additional relevant factor explaining the relationship between availability norms and burnout symptoms. Availability norms heighten the risk of experiencing burnout, not only because they instigate a feeling of pressure but also because they undermine the autonomy that an employee experiences at work. Also this mediational effect was confirmed in this study.

### 5.4. The relationship between telepressure and job autonomy

Following the literature addressing the impact of ICT use outside work hours and the role of social norms herein, we demonstrated that telepressure and job autonomy are both important factors explaining how availability norms heighten the risk of experiencing burnout symptoms. Yet, job autonomy and telepressure are not independent of each other. Barber et al. (2019) specifically showed how telepressure is negatively related to job autonomy. Moreover, the logic behind the autonomy paradox (Mazmanian et al., 2013) involves that ICT developments can undermine autonomy as employees may experience pressure to respond immediately. This seems to imply that job autonomy and telepressure are merely two sides of the same coin. However, the interrelationships among job autonomy and telepressure appear more complex. Telepressure can develop from outside sources, in the form of availability norms that become internalized. But it can also develop from within, as Grawitch et al. (2018) demonstrated that about 40% of explained variance in telepressure was accounted for by personality characteristics like neuroticism. In addition, availability norms are merely one aspect involved in the job autonomy experienced by an employee apart from others, like the nature of the work or the quality of work relationships (Gallie et al., 2004; Esser and Olsen, 2012). Hence, as also confirmed by our data, telepressure and job autonomy are two clearly distinct factors, wherein the former could involve more variance on a personal level and the latter could involve more variance dependent on the specific work context. How telepressure and job autonomy evolve over time in response to different work contexts and different individual characteristics is a promising avenue for future research.

### 5.5. Limitations and future research directions

This study is not without limitations. The first limitation is the cross-sectional design, which makes it difficult to establish causal relationships between the different variables. A longitudinal study would provide more clarity on the strength and direction in which these variables would be related. For example regarding job autonomy, it might be interesting to see how this autonomy evolves over time related to emerging social norms within the team. Moreover, including additional constructs in the analyses would be valuable. For instance, the impact of social norms depends on the degree in which one identifies with the group (White et al., 2009). Hence, the role of social identity could form a relevant avenue for future research. Work-related rumination, or perseverative thinking about work (Querstret and Cropley, 2012), is another concept that might be interesting to study in relation to telepressure in the future. Moreover, as telepressure also depends on individual characteristics (Grawitch et al., 2018), it would be valuable to include these personal characteristics, like self-monitoring or emotional stability, in future analyses. In this sense, “Fear of missing out” (FOMO) could play a moderating role in the relationships presented in this study. Barber and Santuzzi (2017) provided initial evidence for this as they showed that students who score high on telepressure also score high on FOMO. It could be valuable to add this personal characteristic to the analyses.

Secondly, the respondents in this study consisted of a relatively small sample of mainly higher educated Dutch employees. Therefore, the results cannot be generalized to all employees who have ICT tools at their disposal. Yet, we were able to compose a heterogeneous sample with participants covering various sectors and age groups. Nevertheless, future studies should try to deepen our results using larger samples. As the current sample involved a heterogeneous group of respondents from different organizations, it might also be interesting to study the relationship between availability norms and various aspects of occupational health for more specific work situations within one or more specific organizations.

A final limitation of this study concerns the fact that this study took place during the COVID-19 pandemic in 2020. Many employees were required to work from home because of COVID-19 restrictions. This may have influenced the extent to which employees experienced telepressure, job autonomy, or burnout symptoms. However, the questionnaire did include a control question about the degree in which one worked from home. While this control variable showed a significant positive relationship with experiencing burnout symptoms, the effects of availability norms, job autonomy, and telepressure on burnout symptoms remained strongly significant when controlling for working from home. This indicates that the effect of the exceptional pandemic situation on the found relationships might be rather limited.

### 5.6. Conclusion and practical implications

Working in a way that is independent of time and place by using ICT will continue to grow. Our study highlighted that it is not the ICT use outside work hours itself that seems specifically harmful for employee wellbeing, but rather the experienced availability norms regarding this after hours ICT use. These availability norms appeared to instigate pressure to respond immediately and to reduce job autonomy. We add to the literature as we demonstrate that both job autonomy and telepressure play a relevant mediating role in how these norms are associated with heightened feelings of burnout.

This study raises awareness for developing social norms at work that do not pressure employees to send work-related messages in their free time and that support their experience of job autonomy. Our study specifically highlighted that both are important when one wants to reduce the burnout risk at work. Employees who experience a lot of pressure to respond immediately will be at risk of developing burnout feelings, regardless of whether they still think that they can determine their own work approach. Or the other way around, employees who feel their autonomy is undermined will be at risk of experiencing burnout, even when they personally experience little telepressure. Both are needed. Rules and regulations regarding ICT use after hours often focus on reducing pressure, but focusing on respecting autonomy appears equally important. As such, dealing with employees who can work anywhere and anytime requires the right support from management (Ipsen et al., 2022). Based on the insights of this paper, a recommendation would be to give employees the choice in how they want to use the ICT tools at their disposal. After all, some employees might enjoy the evening hours to calmly prepare some e-mails. Yet, at the same time a team leader could communicate repeatedly and consistently that sending e-mails to colleagues outside work hours is not the norm. Overall, it appears valuable to pay careful attention to how norms develop within the organization, respecting employees’ personal choice in using ICT without instigating pressure to respond immediately. No pressure and personal choice appear key.

## Data availability statement

The datasets presented in this study can be found in online repositories. The names of the repository/repositories and accession number(s) can be found at: https://doi.org/10.17026/dans-z9b-7fej.

## Ethics statement

The studies involving human participants were reviewed and approved by Ethics Committee of Open Universiteit. The patients/participants provided their written informed consent to participate in this study.

## Author contributions

KH, JR, KP, and SL contributed to the concept and design. SL contributed to the data collection under supervision of JR and KP. KH and JR contributed to the data analyses. KH contributed to writing the manuscript. All authors contributed to the article and approved the submitted version.

## Funding

The APC was funded by Library Committee Open Universiteit—OA Fund.

## Conflict of interest

The authors declare that the research was conducted in the absence of any commercial or financial relationships that could be construed as a potential conflict of interest.

## Publisher’s note

All claims expressed in this article are solely those of the authors and do not necessarily represent those of their affiliated organizations, or those of the publisher, the editors and the reviewers. Any product that may be evaluated in this article, or claim that may be made by its manufacturer, is not guaranteed or endorsed by the publisher.
